# Comparing the Predictive Value of SOFA and SIRS for Mortality in the Early Hours of Hospitalization of Sepsis Patients: A Systematic Review and Meta-analysis

**DOI:** 10.34172/aim.28567

**Published:** 2024-08-01

**Authors:** Mahdi Majidazar, Farzaneh Hamidi, Nazanin Masoudi, Zahra Vand-Rajabpour, Seyed Pouya Paknezhad

**Affiliations:** ^1^Emergency and Trauma Care Research Center, Tabriz University of Medical Sciences, Tabriz, Iran; ^2^Department of Biostatistics, Faculty of Medical Sciences, Tarbiat Modares University, Tehran, Iran; ^3^Department of Statistics and Epidemiology, Faculty of Health, Tabriz University of Medical Sciences, Tabriz, Iran

**Keywords:** Critical care, Infection, Morbidity, Mortality, Predictive Value, Sepsis, Systematic review

## Abstract

**Background:**

Sepsis, a deadly infection causing organ failure and Systemic Inflammatory Response Syndrome (SIRS), is detected early in hospitalization using the SIRS criteria, while sequential organ failure (SOFA) assesses organ failure severity. A systematic review and meta-analysis was evaluated to investigate the predictive value of the SIRS criteria and the SOFA system for mortality in early hospitalization of sepsis patients.

**Methods:**

Inclusion criteria were full reports in peer-reviewed journals with data on sepsis assessment using SOFA and SIRS, and their relationship with outcomes. For quality assessment, we considered study population, sepsis diagnosis criteria, and outcomes. The area under the curve (AUC) of these criteria was extracted for separate meta-analysis and forest plots.

**Results:**

Twelve studies met the inclusion criteria. The studies included an average of 56.1% males and a mean age of 61.9 (±6.1) among 32,979 patients. The pooled AUC was 0.67 (95% CI: 0.60-0.73) for SIRS and 0.79 (95% CI: 0.73-0.84) for SOFA. Significant heterogeneity between studies was indicated by an I2 above 50%, leading to a meta-regression analysis. This analysis, with age and patient number as moderators, revealed age as the major cause of heterogeneity in comparing the predictive value of the SOFA score with SIRS regarding the in-hospital mortality of sepsis patients (*P*<0.05).

**Conclusion:**

The SOFA score outperformed the SIRS criteria in predicting mortality, emphasizing the need for a holistic approach that combines clinical judgment and other diagnostic tools for better patient management and outcomes.

## Introduction

 In the 21st century, there have been several outbreaks of severe infectious diseases, including SARS in 2003, swine flu in 2009, and COVID-19 in 2020. These outbreaks have had a significant impact on global health. At the same time, non-communicable diseases are responsible for over 35 million deaths annually, accounting for nearly two-thirds of all deaths worldwide. Most of these mortalities are observed in countries classified as low- to middle-income, and these pathologies lead to considerable economic expenditures.^1^

 In addition, the high incidence of infectious diseases can have devastating effects on communities, including death, hospitalization, and severe complications. “Sepsis”, a critical condition triggered by the body’s immune response to infection, stands as one of the most fatal infectious diseases. According to the statistics from the Centers for Disease Control and Prevention (CDC), annually 1.7 million individuals in the United States are diagnosed with sepsis, of whom almost 270 000 progress to death.^2^

 Based on comprehensive epidemiological research encompassing 6 million individuals, the occurrence rate of sepsis is 3 instances per 1000 individuals annually, leading to an estimated 750 000 cases each year in the United States. With an estimated annual mortality rate of 30 to 50 deaths per 100 000 population, sepsis is among the top 10 causes of death. The disease affects individuals of all ages and genders, ranging from mild symptoms to organ dysfunction and shock.^3^

 Sepsis is a form of inflammation instigated by the body’s immunological reaction to an infection. The infection can originate from various sources such as bacteria, exemplified by methicillin-resistant *Staphylococcus aureus* (MRSA), in addition to fungi, viruses, or parasites. The disease frequently affects organs such as the lungs, brain, urinary tract, skin, and abdominal organs. Manifestations of sepsis encompass fever, elevated heart rate, and increased respiratory rate, which may present as pneumonia or painful urination in the case of a kidney infection.^4^

 Severe sepsis can result in organ failure, and one of the most serious complications is systemic inflammatory response syndrome (SIRS), which is inflammation that affects most body organs. SIRS can manifest as an infectious or non-infectious invasion and causes widespread inflammation, leading to failure and disturbances in the body’s systems. This syndrome is a cytokine release syndrome, during which the regulatory system of certain cytokines is disrupted.^5,6^

 Since the mid-1980s, SIRS has been utilized for the prompt detection of sepsis. However, the diagnostic criteria for SIRS are considered “non-specific” and must be interpreted and applied based on the clinical situation and conditions. Despite some objections to these criteria, they are still widely used in clinical practice in many countries.^7^

 In 2016, the sequential organ failure assessment (SOFA) criteria were suggested to evaluate the severity of organ failure.^8^ SOFA, initially introduced in 1994, is an index for assessing organ failure that includes six organs: the lungs, blood, cardiovascular system, liver, central nervous system, and kidneys. This tool systematically and continuously evaluates the condition of these organs during a patient’s hospitalization in the intensive care unit.^9^

 SOFA evaluates parameters related to five vital organs: the lungs, heart and blood vessels, liver, kidney, and central nervous system. The scoring system ranges from normal to abnormal, with a normal condition receiving a score of zero and an abnormal condition receiving a score of four.

 The utilization of these two criteria during the initial hours of hospitalization, frequently termed as the “golden time,” is pivotal for the early detection and management of sepsis, which can substantially enhance patient outcomes. Nevertheless, it is imperative to underscore that while these tools provide valuable insights, they are most effective when used in synergy with clinical judgment and additional diagnostic instruments, thereby ensuring a holistic evaluation of the patient’s condition. This comprehensive approach is crucial for devising an effective treatment strategy tailored to the patient’s specific needs.^10,11^

 Many clinicians use both the SOFA and SIRS criteria to predict the mortality of sepsis and severe sepsis. Timely intervention is crucial for these patients to prevent increased costs, extended hospital stays, organ failure, and death. Predictive scoring systems like SOFA can greatly aid in the clinical evaluation of disease severity and the prediction of patient mortality risk.

 Given the importance of predicting mortality based on the SIRS criteria and the SOFA scoring system and the need for immediate antibiotic treatment after diagnosis, a meta-analysis study was conducted to measure the predictive value of these two systems in terms of in-hospital mortality among sepsis patients. The main objective of this study was to evaluate the prognostic accuracy of the SOFA score in comparison to the SIRS criteria for predicting mortality rates in hospitalized patients with sepsis.

## Materials and Methods

###  Study Design

 Our research was executed as a systematic review and meta-analysis.

###  Search Strategy

 A systematic and comprehensive search of ProQuest, SCOPUS, Web of Science and PubMed, from December 2009 to December 2022, was applied to compare the predictive value of the SOFA score with SIRS for in-hospital mortality of patients with sepsis. The SOFA and SIRS criteria were established in 1996 and 1991, respectively. However, our literature search, which used databases such as Google Scholar and PubMed, revealed that studies comparing these two criteria began to appear around 2009. These studies investigated and reported on the comparative results of the SOFA and SIRS criteria. Consequently, we initiated our source search in December 2009.

 The search strategy was designed with specific keywords as mentioned in following: ((((“Sepsis”[Mesh]) OR (((((((Sepsis[Title/Abstract]) OR (Pyemia*[Title/Abstract])) OR (Pyohemia*[Title/Abstract])) OR (Pyaemia*[Title/Abstract])) OR (Septicemia*[Title/Abstract])) OR (“Blood Poisoning*”[Title/Abstract])) OR (“Bloodstream Infection*”[Title/Abstract]))) AND ((“Systemic Inflammatory Response Syndrome”[Mesh]) OR ((SIRS[Title/Abstract]) OR (“systemic inflammatory response syndrome*”[Title/Abstract])))) AND ((“Mortality”[Mesh]) OR (((mortality*[Title/Abstract]) OR (death[Title/Abstract])) OR (Fatality[Title/Abstract])))) AND ((“Intensive Care Units”[Mesh]) OR ((ICU[Title/Abstract]) OR (“Intensive Care Unit*”[Title/Abstract]))).

## Eligibility Criteria

 We confined our study to scholarly articles in the English language that encompassed both pediatric and adult populations. To uncover further pertinent research, the bibliographies of all qualifying articles and reviews were subjected to thorough examination. Given our familiarity with the existing literature, we projected that the majority of the studies we identified would employ a cohort methodology. Nevertheless, all the study designs we encountered fell within either a cohort or retrospective framework.

 Our study inclusion criteria encompassed the following aspects: 1) comprehensive reports published in peer-reviewed scientific journals, 2) data detailing the evaluation of sepsis using both the SOFA and SIRS criteria, and 3) an examination of the correlation between sepsis screening criteria and reported outcomes, specifically the area under the curve (AUC) along with upper and lower confidence intervals. The primary outcome of interest was mortality, occurring either during hospitalization or upon discharge.

**Figure 1:**
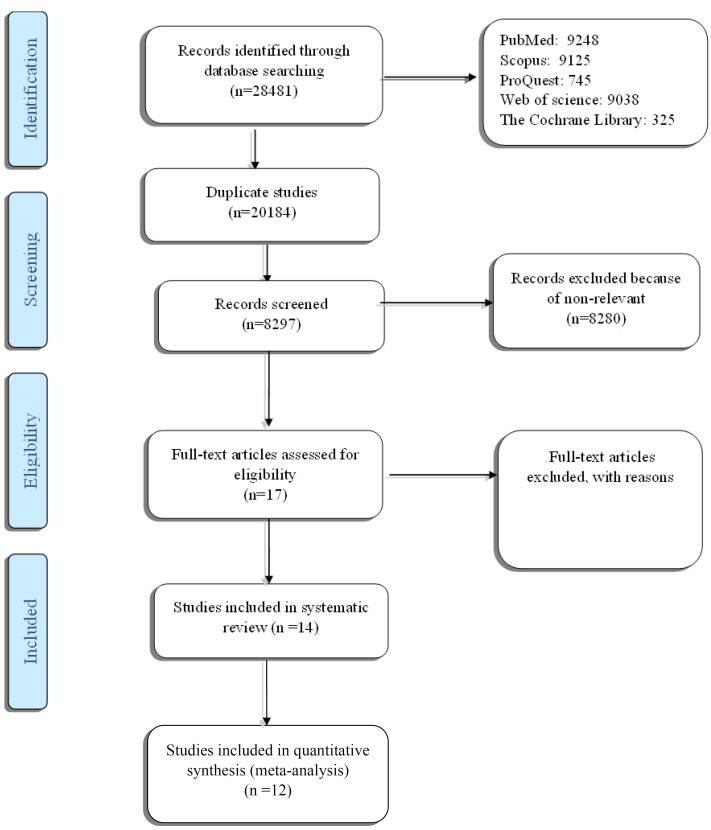
Flow Diagram of the Search Strategy

 Upon final review of the collected articles, two were excluded from the study due to their focus on pediatric sepsis. Additionally, three articles were deemed unsuitable for inclusion owing to insufficient data. Finally, 12 articles were in line with the inclusion criteria and were selected. Figure 1 illustrates the flow diagram of the study’s search and selection process, adhering to the PRISMA methodology.

###  Data Collection and Analysis

 Three researchers (F. H., M. M., and S. P. P.) conducted the study selection procedure, which encompassed the preliminary literature search for identification of references and the subsequent evaluation of potentially relevant titles based on abstracts. This process extended to the review of full-length reports, with all selections being made through consensus. Figure 1 presents the details of the search and study selection process.

###  Risk of Bias

**Figure 2:**
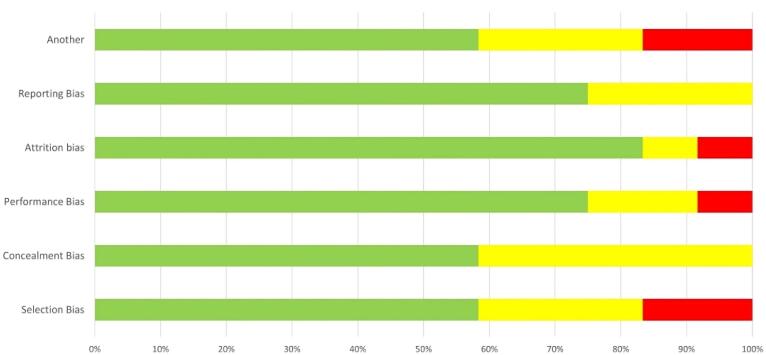
Risk of Bias for Studies of Overall Prognosis

 The risk of bias was assessed using the Cochrane Collaboration tool,^12^ which includes six domains, as illustrated in Figure 2. In line with this, every domain was evaluated and categorized as having a high, unclear, or low likelihood of bias.

###  Data Extraction and Quality Assessment 

 Two authors (F. H. and M. M.) independently conducted data extraction from the chosen articles. The recorded data included study characteristics (such as study type, patient selection criteria, enrollment numbers), patient demographics (age, sex), and outcomes (specifically, hospital mortality or discharge status). In order to evaluate quality, we extracted data about the study group, the criteria for diagnosing sepsis, and the results, which can be found in Table 1.

**Table 1:** Characteristics of the Included Studies

**Study/year**	**Patient, no**	**Sex (Male%)**	**Age**	**AUC of SIRS (Lower, Upper CI)**	**AUC of Sofa (Lower, Upper CI)**
Raith et al, 2017^13^	1184575	55.4	62.9	0.691 (0.688-0.694)	0.76 (0.758-0.764)
Rosa et al, 2017^14^	1487	-	-	0.64 (0.62-0.67)	0.64 (0.62-0.67)
Solligård and Damås, 2017^15^	148570		62.9	0.589 (0.588-0.593)	0.753 (0.750-0.757)
Costa et al, 2017^16^	450	55.1	59.6	0.62 (0.56- 0.67)	0.76 (0.71-0.81)
Khwannimit et al, 2018^17^	2350	56.1	62	0.587(0.566-0.608)	0.839 (0.823-0.855)
Schlapbach et al, 2018^18^	2594	-	-	0.727 (0.682- 0.772)	0.829 (0.791-0.868)
Gaini et al, 2019^19^	323	51	66	0.62 (0.49-0.74)	0.83 (0.76-0.9)
Wu et al, 2019^20^	1831	62.6	-	0.698 (0.657-0.739)	0.771 (0.732-0.81)
Koch et al, 2020^11^	13780	-	64	0.54 (0.53-0.55)	0.73 (0.7-0.77)
Abdullah et al, 2021^21^	2045	48.60	73.2	0.52 (0.47-0.56)	0.688 (0.64-0.73)
Peng et al, 2021^10^	431	58.5	53.1	0.935 (0.907-0.956)	0.973 (0.953-0.986)
Gao et al, 2022.^22^	707	62.1	53.6	0.838 (0.742-0.943)	0.866 (0.779-0.954)

###  Analytical Approach

 We assessed patient attributes, the diagnosis of sepsis (with AUC and corresponding confidence intervals), and patient outcomes (hospital mortality or discharge) based on the SIRS and SOFA criteria.

 We conducted a comparative study to assess how effectively the SIRS and SOFA criteria predict mortality in cases of sepsis. For the robustness of the relationship between sepsis diagnostic criteria and mortality, we utilized the area under the receiver operating characteristic curve (AUROC), along with 95% confidence intervals. We opted for AUROC as the effect measure for the outcome (death) in our meta-analysis due to its reduced susceptibility to artificial inflation arising from heterogeneity, in contrast to risk difference. Furthermore, AUROC was consistently reported across most studies. We employed the I2 test in our meta-analysis to quantify the percentage of total variation across the study estimates which is attributable to heterogeneity. Publication bias was assessed using a funnel plot and trim-and-fill method. We performed all analyses using STATA, version 18.^23^

###  Meta-Regression

 Meta-regression,^24^ involves using either a fixed effects model or a random effects model, where one or more characteristics of the studies are included as covariates. In a fixed effects meta-regression model examining the impact of y, it is represented as follows:


$$f_{i}\left(x,y\right)=\alpha_{i}+\beta_{i}x+\gamma y,$$

where *γ* is the common effect of covariate *y*, a random-effects meta-regression model is given by:


$$f_{i}\left(x,y\right)=\alpha_{i}+\beta x+z_{i}x+\gamma y.$$

A test assessing the null hypothesis γ = 0 aims to ascertain if the covariate y contributes to the variability observed in the study. Meta-regression streamlines the testing process by incorporating all included studies, thereby reducing the number of tests and estimations required, in contrast to subgroup analysis.^25^ As a result, the analysis has higher statistical power and a decreased likelihood of false-positive results. However, it is essential to limit the number of covariates included in a meta-regression and specify them in the systematic review protocol.^26^

## Results

 The preliminary search found 28 481 cited articles. Duplicate articles were eliminated, leaving 17 articles for detailed review after evaluating their abstracts. In cases where there were discrepancies (n = 5) between the two evaluators, consensus was reached through further discussion. In the end, 12 studies fulfilled the selection criteria and were chosen. The PRISMA methodology’s process flow diagram, which illustrates the search and selection of studies, is shown in Figure 1.

 The assessment of each study’s risk of bias across different domains is depicted in Figure 2. All studies were deemed to have a low risk of bias. Notably, approximately 80% of the included studies were evaluated as having a low risk of bias regarding study attrition. In total, 60% of the studies were classified as having low risk, 25% as moderate risk, and 15% as high risk (Figure 2).

 Details regarding the characteristics of the 12 selected studies are outlined in Table 1. Two of the studies (Schlapbach et al in 2018 and Wu et al in 2019) about children remained studies were about adults. On average, the percentage of males was 56.1% and the mean ( ± SD) age was 61.9 ( ± 6.1) out of a total of 32 979 patients who were evaluated. The design of all studies was cohort except for that of Gaini et al in 2019, which was retrospective. In each study, the AUCs of the SIRS and SOFA criteria were extracted separately. Based on the aim of the study which means comparing the prediction of the two criteria, we obtained a separate meta-analysis and forest plot.

###  Overall Analysis of the AUC 

**Figure 3:**
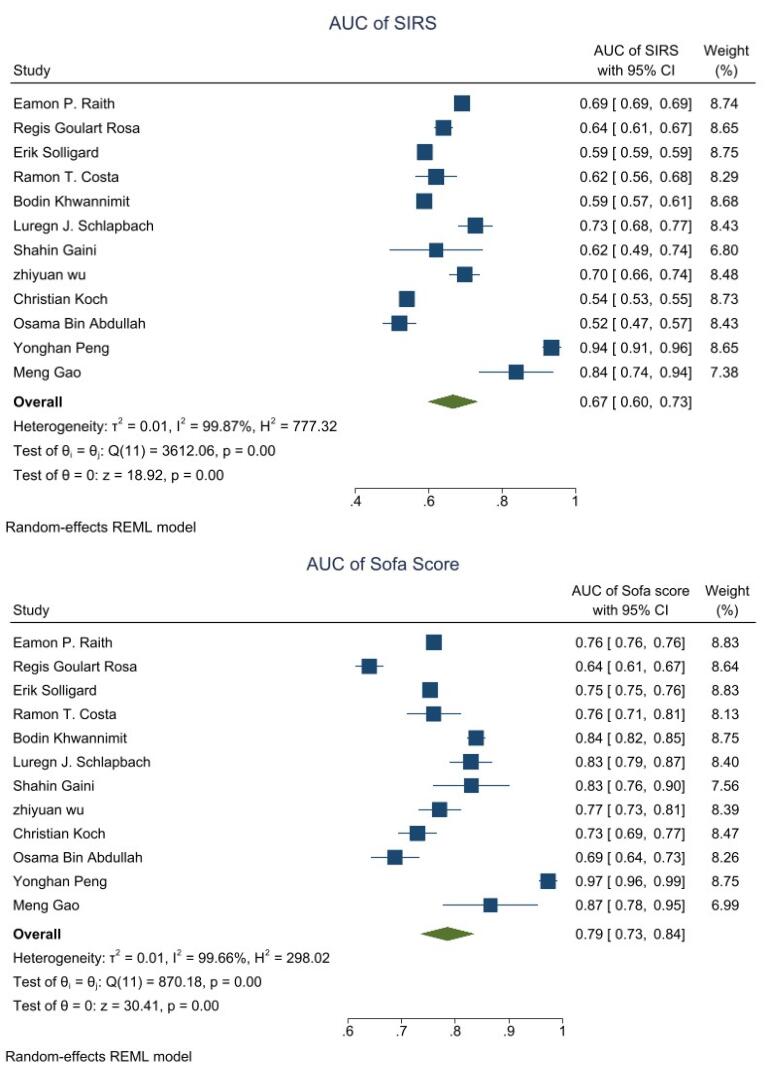
Forest Plot of AUC for Diagnosis of Sepsis Using the SOFA and SIRS Criteria. Analysis model = random effect, statistical method = I^2^

Figure 3 shows that the pooled AUC was 0.67 (95% CI: 0.60-0.73) for SRIS and 0.79 (95% CI: 0.73-0.84) for SOFA. In general, I^2^ above 50% indicates the presence of significant heterogeneity between studies. To address this problem, we proceeded to meta-regression analysis.

###  Meta-Regression Analysis

 Meta-regression analysis was calculated by entering age and patient number as moderators in the association with AUC and SIRS or SOFA high heterogeneity. On univariate meta-regression results are presented in Table 2. Only the age factor was the major cause of heterogeneity in the analysis of comparing the predictive value of SOFA score with SIRS for in-hospital mortality of patients with sepsis (*P *value < 0.05).

**Table 2:** Univariate Meta-regression Analysis of Potential Sources of Heterogeneity on Comparing the Predictive Value of SOFA Score with SIRS for In-hospital Mortality of Patients with Sepsis

**Model**	**Covariate **	**Coefficient **	***P *** **Value**	**R-squared (%)**
SOFA score	Intercept	1.53	0.00^*^	65.10
	Age	-0.01	0.00^*^	
	Patient number	-2.50e-07	0.276	
SIRS score	Intercept	1.87	0.00^*^	62.07
	Age	-0.019	0.00^*^	
	Patient number	-10.24e-08	0.975	

*Statistically significant.

## Discussion

 Sepsis is a lethal disease that results from the body’s overly intense reaction to an infection. The inflammation can spread throughout the body, and in severe cases, can lead to septic shock, death, or disability. Many doctors use two criteria, Sofa and SIRS, to predict sepsis and severe sepsis mortality.^27^

 Considering the importance of this issue (predicting mortality based on the SIRS criteria and SOFA scoring system and the importance of starting antibiotic treatment immediately after diagnosis, especially in the first hour), the aim of this study is to evaluate the predictive value of these two systems regarding the hospital mortality of sepsis patients. According to the results of a review and meta-analysis of data, the overall estimate of the pooled area under the ROC curve (AUC) was 0.67 (95% CI: 0.60-0.73) for SIRS and 0.79 (95% CI: 0.73-0.84) for SOFA. In 2017, Raith et al conducted a study to validate and assess the increase of SOFA and SIRS scores by 2 or more among critically ill patients with suspected infection. The primary outcome of the study was in-hospital mortality or stay in the intensive care unit for more than three days. Among 184,875 patients with an average age of 62.9 years, 18.7% (34,578) died in the hospital, while 55.7% (102 976) died or experienced three or more days in the intensive care unit. SOFA scores increased by 2 or more in 90.1% of patients, while SIRS scores increased by 2 or more in 86.7% of patients. The study found a significant difference between the SOFA and SIRS criteria for in-hospital mortality, consistent with our findings.^13^

 Moreover, in a study conducted by Solligård and Damås in 2017, the predictive power of the SOFA and SIRS criteria among patients hospitalized in the intensive care unit was compared. The study aimed to assess the external validation and degree of differentiation of three criteria (SOFA, SIRS, Qsofa) in predicting mortality in a hospital among patients suspected of infection. The results showed that among 184,875 patients (mean age, 62.9 years; the most common diagnosis, bacterial pneumonia in 17.7%; and in-hospital mortality, 18.7%), the SOFA score was more significant than 2 in 90.1% of patients and indicated that the diagnostic and predictive value of the SOFA criteria is higher than the SIRS criteria.^28^

 Based on the results of the previous and the present study, the SOFA criteria have higher accuracy in predicting mortality among patients suspected of infection compared to the SIRS criteria.

 As previously highlighted, due to its critical significance, extensive research has been dedicated to this topic in recent years. In 2021, Kilinc Toker et al conducted a retrospective study spanning five years, involving 976 sepsis patients (with a mean age of 72.5 ± 13.7 years, 52.7% female). This investigation represents one of the most recent contributions to the field. Among all sepsis-diagnosed patients admitted to the emergency department, 37.4% required hospitalization, and within this subgroup, 52.3% experienced mortality. Notably, patients presenting with qSOFA (Quick Sequential Organ Failure Assessment) and qSOFA + L criteria ≥ 2 upon arrival at the emergency department exhibited a higher mortality rate. Importantly, this study did not identify statistically significant differences in terms of SIRS, qSOFA, or qSOFA + L criteria among patients who succumbed during their hospital stay.^29^

 As in previous studies and the present study, the SOFA score of AUC (0.89) was remarkably distinctive in predicting sepsis.

 Numerous studies have been conducted to evaluate the predictive validity of two criteria in sepsis patients. This period is critical for several reasons. Firstly, early identification of sepsis is crucial. Despite the advent of modern treatment protocols, the mortality rate associated with sepsis remains significantly linked to delays in appropriate treatment. Secondly, the prognostic capabilities of the SOFA and qSOFA (quick SOFA) scores are noteworthy. They exhibit superior prognostic capabilities for poor clinical outcomes compared to the identification of the condition. Lastly, the mortality rates can be significantly impacted by each hour of delayed treatment.^30-32^ Given the significance of these factors, this article has been updated to underscore the importance of employing these two criteria in patients with septicemia.

 As previously pointed out, sepsis refers to the failure of vital organs following the response to an infection in the patient’s body. The SIRS criteria and SOFA criteria are two of the most important and practical criteria for the timely diagnosis of sepsis in patients who are in emergency and hospital triage. Use of criteria with higher predictive power plays a significant role in the prognosis of patients. In this meta-analysis, which reviewed articles published between 2017 and 2021, the SOFA criteria were found to have higher value and power in predicting the mortality of patients with sepsis.

 Based on the results obtained from this study, it is suggested to prioritize the use of the SOFA criteria over other prediction criteria such as SIRS for patients with sepsis. Additionally, the results of this study highlight the necessity and importance of the SIRS criteria and SOFA criteria for sepsis patients.

 Moreover, out of the 12 studies that were extracted, only two were related to children. Additionally, most of the studies were conducted among middle-aged patients. Hence, it is recommended that forthcoming research should focus on assessing children and patients with a younger average age.

## Conclusion

 The aim of this study was to evaluate the predictive accuracy of the SOFA and SIRS criteria during the early stages of hospitalization, given the critical role of sepsis assessment upon admission to the emergency room. A meta-analysis was performed, examining data from studies published between 2017 and 2021. The results of this analysis allowed for evaluation of the predictive efficacy of these two criteria. The findings suggest that the SOFA criteria exhibit superior predictive power in forecasting mortality among septic patients. Despite the demonstrated superior predictive value of the SOFA score compared to the SIRS score in determining mortality among sepsis patients during the initial hours of hospitalization, it is crucial to consider other factors. These include clinical judgment and additional diagnostic tools, which should be integrated into a comprehensive approach for the evaluation and management of these patients. This holistic approach ensures a more accurate and effective treatment strategy, ultimately improving patient outcomes.
